# Detecting Avian Influenza Virus (H5N1) in Domestic Duck Feathers

**DOI:** 10.3201/1410.080415

**Published:** 2008-10

**Authors:** Yu Yamamoto, Kikuyasu Nakamura, Masatoshi Okamatsu, Ayako Miyazaki, Manabu Yamada, Masaji Mase

**Affiliations:** National Institute of Animal Health, Tsukuba, Japan

**Keywords:** Ducks, feathers, H5N1 subtype, influenza A virus, letter

**To the Editor:** Free-range domestic ducks can be a key factor in regional spreading of Asian subtype H5N1 avian influenza (AI) virus (*1*–*3*). Even asymptomatic domestic ducks can shed the virus continuously from the oral cavity and cloaca (*3*–*5*). Therefore, early detection of infected ducks that are shedding the virus would reduce the risk of spreading AI virus (H5N1) in a region where the virus has been endemic in domestic ducks. We previously reported that AI virus (H5N1) can replicate in feather epidermal cells in asymptomatic domestic ducks (*6*). Feathers are living tissues that are easily collectible from live birds with minimal damage. We now report the usefulness of feathers for virus detection in domestic ducks.

An experimental infection study was conducted with Japanese domestic ducks (*Anas platyrhynchos* var. *domestica*) and influenza A virus (H5N1) A/chicken/Miyazaki/K11/2007 as previously described (*6*). Three 4-week-old domestic ducks (a–c) were inoculated intranasally with 10^7^ 50% egg infectious doses (EID_50_) of 0.1 mL. All experimental procedures were approved by the Ethics Committee of the National Institute of Animal Health in Japan.

Inoculated ducks did not show any clinical signs except for persistent corneal opacity on day 3 or later. We collected 3–5 contour feathers, plucked from the body, and 2 sets of oropharyngeal and cloacal swabs from each duck at 24-hour intervals from days 2 through 10 postinoculation (pi). Samples were examined by rapid tests, virus isolation, and reverse transcription–PCR (RT-PCR). Feathers were also examined by immunohistochemical testing.

On-site rapid tests were performed with a commercial kit, QuickVue Influenza A+B (Quidel Corp., San Diego, CA, USA), which can detect influenza virus nucleoprotein. The first set of swabs was used for rapid tests according to the manufacturer’s instructions. We also tested 1–2 sticks of the feather calamus (≈15–30 mg per stick) for rapid tests (Appendix Figure, panel **A**). Briefly, we put the calamuses into the test tube containing attached reagent solution (340 μL) and chopped them into small pieces with an iris scissor and then placed the test strip in the tube. We obtained the following results: feathers tested positive for influenza A virus from days 3 through 6 pi in 1 duck (a), and on days 3 and 4 pi in 2 ducks (b and c), whereas all oropharyngeal and cloacal swabs were negative (Appendix Figure, panel **B**).

For virus isolation, we used a second set of swabs placed in 1 mL of phosphate-buffered saline containing antimicrobial drugs and the remaining 2–3 feather calamuses. Virus titers of swabs and 10% (wt/vol) feather homogenate supernatants were calculated with 10-day-old embryonated chicken eggs and expressed as EID_50_/mL. Viruses were isolated from the oropharyngeal swabs, cloacal swabs, and feathers of all birds, and feathers tested positive for the virus for a longer period than did the swabs (Table). Although the feather samples used for virus isolation differed from those used in rapid tests, the higher virus titers of feathers in each bird corresponded to the positive period of feathers for rapid tests.

**Table:** Results of virus isolation and RT-PCR in 3 domestic ducks inoculated with influenza A virus (H5N1)*

dpi	Virus titer (RT-PCR result)†										
	Duck a				Duck b				Duck c		
	F	O	C		F	O	C		F	O	C
2	4.2† (+)‡	2.0 (+)	– (–)		1.7 (+)	1.7 (+)	– (+)		2.8 (+)	2.0 (+)	1.7 (–)
3	6.9 (+)	2.7 (+)	– (–)		4.5 (+)	3.0 (+)	2.5 (+)		4.8 (+)	1.7 (+)	2.8 (–)
4	6.8 (+)	3.7 (+)	1.7 (–)		2.3 (+)	3.5 (+)	1.7 (–)		4.8 (+)	3.3 (+)	– (–)
5	6.5 (+)	– (+)	– (–)		1.7 (+)	– (+)	– (–)		2.5 (+)	1.7 (+)	– (–)
6	5.6 (+)	– (+)	– (–)		– (–)	– (–)	– (–)		4.0 (+)	– (–)	– (–)
7	3.8 (+)	– (–)	– (–)		– (–)	– (–)	– (–)		– (–)	– (–)	– (–)
8	5.8 (+)	– (–)	– (–)		– (–)	– (–)	– (–)		– (+)	– (–)	– (–)
9	– (–)	– (–)	– (–)		– (–)	– (–)	– (–)		– (–)	– (–)	– (–)
10	– (–)	– (–)	– (–)		– (–)	– (–)	– (–)		– (–)	– (–)	– (–)

*RT-PCR, reverse transcription–PCR; dpi, days postinoculation; F, feathers; O, oropharyngeal swabs; C, cloacal swabs. †Virus titer expressed as 50% egg infectious doses per mL; –, negative for virus isolation. RT-PCR result: +, positive; –, negative.

One-step RT-PCR was performed on the total RNA extracted from the same samples as in virus isolation to detect the H5 AI virus gene (SuperScript One-Step RT-PCR System; Invitrogen, Carlsbad, CA, USA). The 1:10 dilution of RNA templates was used for feathers. The primers used were H5–248–270F and H5–671–647R; the expected product was 424 bp (*7*). The sensitivity of RT-PCR was slightly higher than that of virus isolation except for the results with cloacal swabs (Table).

Immunohistochemical testing was performed to detect influenza virus nucleoprotein in the feather tissue by using a rabbit polyclonal antibody (ab22285; Abcam Ltd., Cambridge, UK). Virus antigens were detected in feather epidermal cells from days 3 through 6 pi, and in a few stromal cells in the feather pulp on days 3 and 4 pi (Appendix Figure, panel **C**).

Our results indicate that larger amounts of viruses can be isolated for a longer time from feathers than from swabs. Therefore, feathers can be considered useful samples for surveillance or diagnostic examination of AI virus (H5N1) in domestic ducks. The epidermis, the outer layer of the feather, is a tissue that has poor host immune response against viral replication (*8*). As has been observed in virus isolation, viruses may be able to survive longer in differentiated epidermal tissue such as contour feathers.

The sensitivity of the rapid test was not adequate for swabs, a finding similar to that of other studies (*9*,*10*). However, positive results for rapid tests of feather samples only may shed light on the on-site field detection of AI (H5N1) in asymptomatic domestic ducks. When virus shedding from domestic ducks is maintained at a low level of viral load during the infection, selecting the sample with higher viral load and antigens in tissues, such as feathers, can increase the detection rate obtained from on-site examination. Our results show the potential of feathers as candidates for early AI virus (H5N1) detection.

## Supplementary Material

Appendix Figure A) Developing contour feather. The calamus was used for examination (bar = 1 cm). B) Result of the rapid test with feathers. A pink line (arrowhead) indicates a positive result for influenza A virus. C) Immunohistochemical stain of a biopsied feather composed of feather epidermis (fe) and feather pulp (fp). Influenza virus nucleoprotein was detected in the fe epidermal cells (arrowheads) (bar = 200 m).
