# Supplementary figures and images for: Detecting Avian Influenza Virus (H5N1) in Domestic Duck Feathers
Source: Emerg Infect Dis. 2008 Oct;14(10):1671–2. doi: 10.3201/1410.080415 (PMC2609858; doi:10.3201/1410.080415)

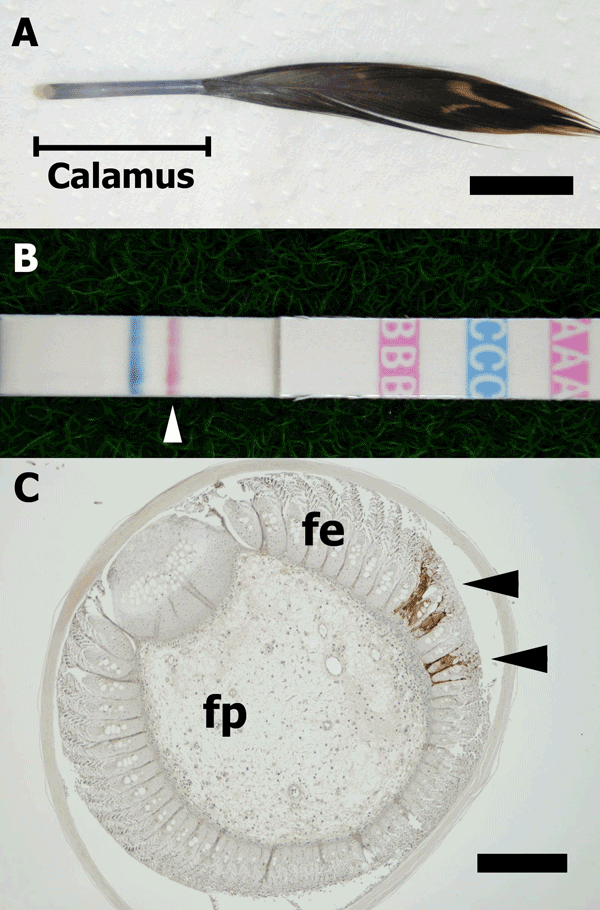

Supplement: Appendix Figure — A) Developing contour feather. The calamus was used for examination (bar = 1 cm). B) Result of the rapid test with feathers. A pink line (arrowhead) indicates a positive result for influenza A virus. C) Immunohistochemical stain of a biopsied feather composed of feather epidermis (fe) and feather pulp (fp). Influenza virus nucleoprotein was detected in the fe epidermal cells (arrowheads) (bar = 200 m). [file 08-0415_app-s1.gif]
